# DEVELOPING A SUMMER RESEARCH INSTITUTE FOR EARLY CAREER RESEARCHERS

**DOI:** 10.1093/geroni/igae098.1120

**Published:** 2024-12-31

**Authors:** Chelsea Kline, Sheryl Zimmerman, Julie Bynum, Matthew Baumgart, Sam Fazio

**Affiliations:** Alzheimer’s Association, Chicago, Illinois, United States; University of North Carolina at Chapel Hill (UNC), Chapel Hill, North Carolina, United States; University of Michigan, University of Michigan, Michigan, United States; Alzheimer’s Association, Chicago, Illinois, United States; Alzheimer’s Association, Chicago, Illinois, United States

## Abstract

Today, there are nearly 7 million Americans living with Alzheimer’s disease and more than 11 million unpaid caregivers. By 2060, the number of Americans living with Alzheimer’s could reach almost 14 million. These statistics highlight the need for early career investigators to join the dementia research workforce and accelerate breakthroughs in the field. To address this need, the Alzheimer’s Association Interdisciplinary Summer Research Institute (AA-ISRI) was created. AA-ISRI was developed as a five-day immersive experience in which 24 competitively-selected, early career investigators attend didactic lectures led by experts in the field, develop research proposals in small groups, and receive one-to-one mentoring. The overall goals of the program include increasing knowledge on the fundamental aspects of dementia and dementia research; increasing connections in the field; and aiding the development of fundable research proposals. This presentation will provide an overview of the AA-ISRI, including information on the goals of the program, how and why the program was developed and structured as it is, session topics, and the recruitment and selection process of AA-ISRI participants.

